# Exploiting Language Variation to Better Understand the Cognitive Consequences of Bilingualism

**DOI:** 10.3389/fpsyg.2018.01686

**Published:** 2018-09-07

**Authors:** Andrea A. Takahesu Tabori, Emily N. Mech, Natsuki Atagi

**Affiliations:** Department of Psychology, University of California, Riverside, Riverside, CA, United States

**Keywords:** bilingualism, bilingual advantage, individual differences, executive function, cognitive control

## Abstract

Within the past decade, there has been an explosion of research investigating the cognitive consequences of bilingualism. However, a controversy has arisen specifically involving research claiming a “bilingual advantage” in executive function. In this brief review, we re-examine the nature of the “bilingual advantage” and suggest three themes for future research. First, there must be a theoretical account of how specific variation in language experience impacts aspects of executive function and domain general cognition. Second, efforts toward adequately characterizing the participants tested will be critical to interpreting results. Finally, designing studies that employ converging analytical approaches and sensitive methodologies will be important to advance our knowledge of the dynamics between bilingual language experience and cognition.

## Introduction

A key tenet in research design is parsimony: to design studies that are as simple as possible. However, complex questions and designs are sometimes oversimplified, more so than parsimony requires. For example, the psychological and language sciences have traditionally looked for unifying principles across groups of people, which has led to questions such as “are bilinguals better at cognitive control than monolinguals?" However, this approach leads to group-level analyses with little regard for meaningful variation *within* each group. Rather than treating variation within groups as noise, perhaps we should *start* by studying that variation. Investigating individual differences is not new in the field of Psychology (e.g., Tyler, 1947; Anastasi, 1958), but consistently applying such an approach may provide clarity to the recent controversy about bilingual benefits (Paap and Greenberg, 2013; Paap, 2014; de Bruin et al., 2015; Valian, 2015; von Bastian et al., 2016; but see Baum and Titone, 2014; Fricke et al., in press) and bring about a more nuanced approach to the field. For instance, in much of the published literature on bilingualism, it is difficult to disentangle true null results from those arising from methodological constraints or inadequate comparisons (for a review, see Laine and Lehtonen, in press). This paper identifies three key themes to guide future research in the field. Specifically, we focus on re-examining the notion of the “bilingual advantage," how we report participant characteristics, and how we might modify research methods and analytical approaches to better account for variability. In particular, we advocate for embracing variability and examining individual differences in bilingual experiences to better understand the cognitive and linguistic consequences of bilingualism. These suggestions take a multidimensional approach on language and have implications for all language researchers.

## On the “Bilingual Advantage”

The “bilingual advantage” was a phrase first used to describe a result in which bilinguals out-performed monolinguals on tasks of cognitive control, and this advantage was theorized to be driven by the bilinguals’ constant need to manage competition from each language (Bialystok, 2001; Bialystok et al., 2004). This result sparked interest in the potential cognitive benefits of bilingualism, motivating studies that compared bilinguals and monolinguals on tasks of cognitive control (e.g., Costa et al., 2008; Poarch and van Hell, 2012). Although an increasing number of studies examined speakers in varied locations, of varied ages, and with varied tasks, the underlying theory remained largely unchanged and did not grow to encompass how variation may impact outcomes. As a result, research addressing the “bilingual advantage” became dichotomized, both in the experimental groups tested (monolinguals vs. bilinguals) and in the possible outcomes (bilingual advantage vs. no advantage). As such, an expectation that bilingualism would have a main effect on cognitive control performance became commonplace. Dichotomizing the groups tested and the possible outcomes has created controversy whenever studies do not demonstrate advantages for bilinguals relative to monolinguals. However, the problem with this logic is that bilingualism is a multidimensional construct (Luk and Bialystok, 2013), and as such, cannot be treated as a categorical variable. To overcome the controversy surrounding the bilingual advantage, it will be important to understand the mechanisms by which aspects of bilingual language experience (e.g., proficiency, literacy, age of acquisition) give rise to cognitive adaptations.

The Adaptive Control Hypothesis proposed by Green and Abutalebi (2013) provided an initial step toward understanding the relation between bilingual language contexts and cognitive changes. According to this hypothesis, variation in dual language contexts constrain which languages a bilingual can use and the degree to which they can switch between their languages. This contextual constraint is thought to impact cognitive control in distinct ways. Critically, bilingual language contexts are proposed to have specific cognitive outcomes – not just a generalized advantage. Thus, depending on the particular outcome that is investigated, there may or may not be differences between bilinguals and monolinguals. Finding a lack of differences between bilinguals and monolinguals (or between different types of bilinguals) is not inherently a problem, but rather, can be explanatory in its fit into the broader theoretical framework. For example, more than one aspect of bilingual language variation may be responsible for effects of bilingualism on cognitive control: there are mixed results reported in the language switching, language control, and cognitive control literature (e.g., Paap et al., 2014, 2017; Verreyt et al., 2016). Mixed results could be due to a variety of factors such as not measuring and accounting for a critical aspect of language experience or other relevant variables that also affect cognitive control (e.g., age; Kousaie and Phillips, 2017). Thus, in addition to the Adaptive Control Hypothesis, it will be critical to further develop theories that make specific predictions regarding how the variation in bilingual language experience may give rise to differences in cognitive control or cognition more generally. Without specifying the underlying mechanism, further attempts to investigate bilingual differences may only contribute to, rather than clarify, the controversy.

Pivoting from testing monolinguals vs. bilinguals to answer a yes or no, advantage or no advantage question to one of “bilingual differences” may create greater insight into the mechanisms underlying the consequences of varied language experience (Bak, 2016). Although the primary source of the controversy of the “bilingual advantage” surrounds research investigating the consequences of bilingualism for executive function, the phrase “bilingual advantage” itself is now used widely. In the decade since the initial report (Bialystok, 2001; Bialystok et al., 2004), a virtual explosion of research has arisen claiming bilingual advantages in domains such as visual discrimination and habituation (e.g., Weikum et al., 2007; Sebastián-Gallés et al., 2012; Singh et al., 2015), communicative development (e.g., Fan et al., 2015; Liberman et al., 2017), novel word learning (e.g., Kaushanskaya and Marian, 2009), episodic memory (e.g., Schroeder and Marian, 2012), and phonetic learning (e.g., Antoniou et al., 2015), to name a few. While many of these studies draw connections to the underlying theory relating bilingual language regulation to cognitive control and executive function, more broadly, the results reported are domain-specific and suggest that the controversy surrounding the “bilingual advantage” may be focused too narrowly. While there are many advantages associated with bilingualism, we argue here that it will be important to redefine the way in which we describe such “advantages” to acknowledge the scope of the observed consequences and to promote a more appropriate approach to generalization across studies.

The controversy surrounding the consequences of bilingualism may provide a set of lessons for the field that extend beyond the studies that have been associated with this issue. The lessons emerging from this controversy have relevance not only for those directly investigating the cognitive consequences of bilingualism, but for language science more broadly. Embracing parsimony in the face of complexity may actually lead to oversimplification that slows progress rather than promoting it. Being specific and intentional about the degree to which we generalize terms across domains will clarify similarities and differences between theories of language and cognition. Additionally, there is a fundamental need to embrace variation, appropriately characterize it, and delineate how differences in experience may have consequences for the mind and brain. The following sections identify and suggest initial steps to move toward these goals by providing insight for characterizing our samples and designing our methodology.

## On Characterizing Our Samples: Bilinguals are a Diverse Group

A critical factor that has been largely overlooked in research on the “bilingual advantage” is that bilinguals – as well as monolinguals – are heterogeneous, with a wide range of language backgrounds and experiences. Though a call for more nuanced characterizations of bilinguals’ diverse language experiences is not new (e.g., Green and Abutalebi, 2013; Kroll and Bialystok, 2013; Luk and Bialystok, 2013; Abutalebi and Green, 2016; Bialystok, 2016; Surrain and Luk, in press; Laine and Lehtonen, in press), the focus of much of the published research remains on differences between bilinguals and monolinguals, with little attention paid to who these bilinguals – or even monolinguals, for that matter – are. Understanding speakers’ diverse language experiences will allow for a more critical investigation of the consequences of different language experiences for the mind and brain, providing insight into the interactions and moderating variables that may be obscuring group-level differences between bilinguals and monolinguals.

Given that bilingualism is a dynamic, multidimensional variable (e.g., Luk and Bialystok, 2013), detailed information about participant background and experiences – both past and present – is critical. Although participant characteristics such as self-rated proficiency, amount of use, and age of acquisition of each language are often provided (for a review, see Surrain and Luk, in press), in what context speakers learned and used each language in the past is typically left undescribed. However, there is evidence that learning to read in the home language affects literacy skills in other languages (e.g., Shanahan and Escamilla, 2009; Sparrow et al., 2014; Shin et al., 2015), suggesting that biliteracy – and likely the language of schooling – may be relevant dimensions to examine in studies of bilingualism and cognition. Additionally, language brokering (i.e., informal translation) experience has been found to affect language processing (e.g., López et al., 2017; López and Vaid, 2018) and conceptual representations (e.g., López and Vaid, 2016), pointing to the importance of understanding not only how much bilinguals have used each language but also for what purpose they have used each language. Such findings shed light on the need to consider past language experiences when examining a “group” as diverse as bilinguals.

Additionally, evidence for the enduring consequences of early language exposure can be found in research on functionally monolingual speakers who were exposed to a language early in life, but due to life circumstances, lost explicit knowledge of that language, and consequently function exclusively in their second language. One group of such “monolinguals” is international adoptees (IA): those who were exposed to one language as children and later lost all contact with and knowledge of this language after relocating permanently into their country of adoption. A number of studies suggest that despite having no functional knowledge in their first language and having spent the majority of their lives speaking another language, IAs show language processing signatures that are more similar to those of bilingual speakers of their lost language and their second language than those of monolingual speakers of their second language (e.g., Pierce et al., 2014, 2015, 2017). Similarly, research on childhood overhearers (i.e., adults who, as children, overheard speech in a language other than their native language) also suggests that despite not having productive knowledge of the language they overhead, overhearers are able to learn aspects of the phonology of that language better than those who were not childhood overhearers of that language (e.g., Au et al., 2002; Knightly et al., 2003). Taken together, these findings suggest that despite discontinued use of an early exposed language, there are fundamental changes in language processing that endure into adulthood. Without adequately characterizing speakers’ language history, there would be an incomplete picture of the story of how experience with multiple languages impact cognition. Although IAs and overhearers are traditionally considered monolinguals, these studies demonstrate that there is significant variation with second language experience within monolinguals that, if studied, can contribute to our understanding of bilingualism more generally.

Objective measures of speakers’ language skills are also needed to not only better characterize bilingual and monolingual samples but also understand the cognitive processes underlying language skill. Objective measures of language proficiency – in addition to self-rated proficiency – should be used and reported to provide a more accurate measure of current language skill (e.g., Tomoschuk et al., in press). Although objective proficiency measures have been found to be correlated with self-rated proficiency (e.g., Marian et al., 2007), the addition of objective measures – especially for aspects of language skill that are particularly relevant for a specific research question – could uncover how bilinguals’ diverse language skills may affect cognition as well. For instance, if productive language skills or vocabulary are important aspects of language skill for a particular study, measures such as picture naming tasks (e.g., Multilingual Naming Test; Gollan et al., 2012) and verbal fluency tasks (e.g., Delis et al., 2001) are relatively simple tasks that can be used to objectively measure productive language skills or vocabulary. Additionally, when such objective proficiency measures are combined with cognitive tasks, we can begin to understand what cognitive processes may underlie different language processes (e.g., Zirnstein et al., 2018) – something that is critical to understand in order to uncover the underlying mechanisms of any bilingual differences in cognition. Moreover, objective proficiency measures also better control for cultural differences in self-ratings of language proficiency (e.g., Hoshino and Kroll, 2008; Tomoschuk et al., in press), particularly when comparing multiple groups of bilinguals (e.g., Japanese-English bilinguals vs. Spanish-English bilinguals). Thus, we recommend that future research incorporate both language history questionnaires that capture self-ratings of language proficiency (e.g., LEAP-Q; Marian et al., 2007) as well as objective measures of speakers’ language skills to more accurately characterize speakers.

Precise descriptions of speakers’ languages, as well as clear definitions for terminology used to describe bilinguals, are also necessary. Although terms such as “native language,” “first language,” and “second language” typically provide information about the order of language acquisition, they are often conflated with other aspects of language skill or status. For instance, these terms may be used to describe a speaker’s language dominance (e.g., “native” or “first” language referring to a speaker’s most dominant language) or whether a specific language is the majority vs. minority language (e.g., “native” or “first” language referring to the majority language in the community and “second language” referring to the minority language in the community). Bilinguals can also differ in the nature of the two languages they speak, where some bilinguals’ languages differ in phonemic inventories, script, syntactic rules, or even in modality. Moreover, given that some bilinguals have two first languages that were acquired simultaneously (e.g., De Houwer, 1990), and some speakers have first languages that they can no longer speak and/or understand (e.g., Pierce et al., 2014), first vs. second languages can be arbitrary labels for some speakers. Relatedly, there is little consensus on the definition of a “native” language, and even monolinguals can vary widely in the skill they have in their one and only language (e.g., King and Just, 1991; Tanner and Van Hell, 2014). There is also evidence that monolinguals’ native language undergoes change when speakers begin to acquire a new language (e.g., Bice and Kroll, 2015), suggesting that the native language is not as stable as once thought. We suggest that future research clearly define terminology used to describe bilinguals and monolinguals so that terms such as “first language” do not conflate order of language acquisition with language skill, status, or other characteristics of bilingual language experience. By both using objective measures of language proficiency and being more precise in our descriptions of speakers’ languages, we may be able to understand how diversity in bilinguals’ language skill and status are reflected in cognition as well.

Further details about participants’ sociolinguistic context would also allow for a deeper understanding of what kinds of speakers were included in a study. Although demographic variables that typically covary with bilingualism – such as socioeconomic status, education, and immigration status – are sometimes reported and/or controlled for in studies (e.g., Morton and Harper, 2007; Carlson and Meltzoff, 2008; Alladi et al., 2013), the context of language use is typically unreported. However, evidence suggests that the larger sociolinguistic context surrounding speakers – both bilingual and monolingual – may affect language and cognition. For instance, a bilingual who speaks a language that is uncommon in their sociolinguistic context would not have as many opportunities to use that language – or switch between their two languages—as a bilingual who speaks a language that is common in their sociolinguistic context. Accordingly, the Adaptive Control Hypothesis (Green and Abutalebi, 2013; Abutalebi and Green, 2016) posits that the ways in which bilinguals use their languages with interlocutors has consequences for language and cognitive control. Indeed, a meta-analysis of studies on the effect of bilingualism on cognition found location-based differences in effect sizes, with effect sizes for studies conducted in Europe being significantly greater than those for studies conducted in the United States and the Middle East (Adesope et al., 2010); such findings suggest that the sociolinguistic contexts within each of these locations may have consequences for the relation between bilingualism and cognition. Moreover, recent evidence from different groups of monolinguals has found that the linguistic diversity of monolinguals’ sociolinguistic context impacts infants’ social learning (Howard et al., 2014) and preschoolers’ language awareness (Atagi, 2018). Altogether, such evidence provides insight into the kinds of language experiences that may be critical when describing research participants. Simply knowing whether individuals are immersed in the first or second language and whether they are proficient or not, is not sufficient. Although it would be ideal to use methods such as daily diaries and speech recorders (e.g., LENA; Xu et al., 2009) to collect detailed information about speakers’ context of language use on a day-to-day basis (which has also been suggested by Laine and Lehtonen, in press), these methods are resource-intensive and can be difficult to collect. Thus, minimally, future research should gather information regarding speakers’ social networks and communities – along with any available corresponding census data on sociolinguistic context – to better capture speakers’ context of language use.

Given the lack of detailed information about participants in the majority of presently published works, it is unsurprising that it is still largely unknown how these different language experiences and skills interact to affect cognition. However, recent research suggests that a complex relation exists between language processing, language regulation, and cognitive control (e.g., Zirnstein et al., 2018). By taking a more nuanced approach to understanding and reporting participants’ language backgrounds, we may begin to uncover why and how variability in language background shapes cognition.

## On Studying Individuals: Individual Differences and Change Over Time

A promising direction for the field is to exploit the variability in both current and previous language experiences by examining individual differences – both longitudinally and cross-sectionally – and by conducting more mechanistic studies. As bilingualism is caused by life circumstances rather than experimental ones, bilingualism research has traditionally involved quasi-experimental designs, which is problematic for establishing causality (for a review, see Laine and Lehtonen, in press). One way to overcome this problem is by conducting longitudinal studies to control random variation across time in order to isolate the effects of bilingualism due to cumulative language experience. Longitudinal designs have proven to be particularly sensitive to the consequences of bilingualism over the course of development. Santillán and Khurana (2017) followed a large sample of children and used Structural Equation Modeling to predict executive function trajectories starting from the children’s entry into the Head Start program until their transition to Kindergarten. The model revealed different trajectories for monolinguals, bilinguals, and learners (i.e., children who were transitioning from monolingual to bilingual classrooms). Children who were bilingual at the beginning of Head Start had the highest executive function performance of the three groups and showed the steepest growth over time. The learners had the lowest performance of all groups but showed more accelerated growth and higher executive function skill at Kindergarten entry compared to their monolingual peers. Longitudinal designs not only reveal that the relation between language and cognition differs across the lifespan, but importantly, they suggest that the effects of bilingual language experience may impact developmental and learning trajectories.

Although longitudinal designs are especially informative, the expense associated with such a design often precludes its feasibility. One way to overcome this problem is to conduct short-term longitudinal studies or lab-based training studies that expose participants to a second language and to examine the neural or behavioral changes that occur as a result of that exposure (e.g., McLaughlin et al., 2004; Osterhout et al., 2008; Hämäläinen et al., 2017) or to ask what kinds of changes predict successful L2 learning (e.g., Prat et al., 2016). Training studies have also been used to examine how particular bilingual language skills such as language–switching might impact cognitive control (Zhang et al., 2015), providing a causal link for the relationship between aspects of bilingual language experience and executive function. Given the greater experimental control afforded by these approaches, we propose that examining individual differences through learning and training studies will make some important contributions to the field of bilingualism.

## Conclusion

The controversy involving the “bilingual advantage” has received a great deal of attention in the field with numerous studies addressing the question, and special issues such as this one dedicated to providing productive future directions. In this article, we suggest that much of the controversy in bilingualism research stems from dealing with the variability in bilingual language experiences inappropriately both at theoretical and methodological levels. To study the consequences of knowing multiple languages in its many forms, we must learn to appropriately measure that variation and design studies that can exploit that variation without confounding it with other factors. First, we suggest that if research findings pose problems for existing accounts, we must actively revise those accounts to accommodate for the complexity of the data. Second, we suggest that sensitively measuring and describing the language histories and skills of participants using behavioral and self-report measures will more accurately allow us to capture the effects of bilingualism. Lastly, we propose that diversifying research design by using more (short or long-term) longitudinal studies and by focusing more on individual differences, we can better evaluate how second language experiences affects cognition while avoiding setbacks of quasi-experimental designs. The recommendations proposed in this paper will enable us to move beyond simple group comparisons and to exploit variation to elucidate the relation among language experience, mind, and brain.

## Author Contributions

Each co-author contributed equally in writing the manuscript and prepared it for publication.

## Conflict of Interest Statement

The authors declare that the research was conducted in the absence of any commercial or financial relationships that could be construed as a potential conflict of interest.
